# Eukaryotic Evolutionary Transitions Are Associated with Extreme Codon Bias in Functionally-Related Proteins

**DOI:** 10.1371/journal.pone.0025457

**Published:** 2011-09-23

**Authors:** Nicholas J. Hudson, Quan Gu, Shivashankar H. Nagaraj, Yong-Sheng Ding, Brian P. Dalrymple, Antonio Reverter

**Affiliations:** 1 Computational and Systems Biology, CSIRO Livestock Industries, Queensland Bioscience Precinct, St. Lucia, Brisbane, Queensland, Australia; 2 College of Information Sciences and Technology, Donghua University, Shanghai, China; University of Illinois, United States of America

## Abstract

Codon bias in the genome of an organism influences its phenome by changing the speed and efficiency of mRNA translation and hence protein abundance. We hypothesized that differences in codon bias, either between-species differences in orthologous genes, or within-species differences between genes, may play an evolutionary role. To explore this hypothesis, we compared the genome-wide codon bias in six species that occupy vital positions in the Eukaryotic Tree of Life. We acquired the entire protein coding sequences for these organisms, computed the codon bias for all genes in each organism and explored the output for relationships between codon bias and protein function, both within- and between-lineages. We discovered five notable coordinated patterns, with extreme codon bias most pronounced in traits considered highly characteristic of a given lineage. Firstly, the *Homo sapiens* genome had stronger codon bias for DNA-binding transcription factors than the *Saccharomyces cerevisiae* genome, whereas the opposite was true for ribosomal proteins – perhaps underscoring transcriptional regulation in the origin of complexity. Secondly, both mammalian species examined possessed extreme codon bias in genes relating to hair – a tissue unique to mammals. Thirdly, *Arabidopsis thaliana* showed extreme codon bias in genes implicated in cell wall formation and chloroplast function – which are unique to plants. Fourthly, *Gallus gallus* possessed strong codon bias in a subset of genes encoding mitochondrial proteins – perhaps reflecting the enhanced bioenergetic efficiency in birds that co-evolved with flight. And lastly, the *G. gallus* genome had extreme codon bias for the Ciliary Neurotrophic Factor – which may help to explain their spontaneous recovery from deafness. We propose that extreme codon bias in groups of genes that encode functionally related proteins has a pathway-level energetic explanation.

## Introduction

With fully sequenced genomes now available for organisms of differing complexity, there is greater opportunity to explore genome to phenome relationships. For example, it has become clear that protein coding sequence contains far more information than merely the encoding of amino acids. This is well illustrated by the phenomenon of codon bias [1], which arises when a given amino acid is preferentially encoded by one of various ‘synonymous’ codons.

Studies on codon bias have typically focused on measuring the extent of codon bias in coding sequence of interest and using this information to make predictions about, or influence, the expression levels of proteins. For example, as a consequence of codon bias the amount of a specific protein produced by an organism can be reduced or increased by the introduction of that organism's un-preferred or preferred codons, respectively, into the corresponding protein coding sequence [2]–[4]. Such studies have generated a suite of tools including the general codon usage analysis (GCUA) [2], the codon adaptation index (CAI) [3] and the interactive codon usage analysis (INCA) [4].

The pathological [5] and evolutionary [6], [7], [8] implications of codon bias have also been explored, although to a much lesser extent than the implications for protein expression. This may be because the genome-wide functional data and accompanying statistical enrichment tools–such as GOrilla analysis [9]–have only become available in the recent post-genomics era.

Apparently silent, synonymous codon changes can clearly affect the speed and efficiency of translation [10] and the abundance of proteins [11]. Translation rate constants play a dominant role in control of protein levels, and protein synthesis consumes more than 90% of cellular energy [12]. Thus it is certainly conceivable that natural selection for codon bias in influential biological processes may have substantial macro evolutionary implications. Perhaps such selection could account for many fundamental changes in phenotype that have not yielded to alternative explanations. Indeed, the explanation for increased phenotypic complexity in eukaryote evolution remains elusive. It does not appear to be attributable to simple measures of genome size–the so-called c-value enigma e.g. [13], or to the total number of proteins–which are as numerous, for example, in the unicellular organism, *Chlamydomonas reinhardtii,* as in the 2,000 cell organism, *Volvox carteri* [14].

To explore the possibility that codon bias may play a role in macroevolution, we set out to characterize the patterns of codon bias on a genome-wide scale and to associate the observations with the phylogenetic position of the organisms on the Eukaryotic Tree of Life. Seeking to shed light on the protein-based origins of multicellularity, emergence of the vertebrates, the evolution of mammals and of cognition, we analyzed the following representative organisms: *A. thaliana* (Arabidopsis), *S. cerevisiae* (a yeast), *Caenorhabditis elegans* (roundworm), *G. gallus* (chicken), *Pan troglodytes* (chimpanzee) and *H. sapiens* (human).

We identified a number of new relationships that are challenging to rationalize at the level of the individual gene, given that (with the exception of multigene families) individual genes are generally presumed to have long independent evolutionary histories and yield mRNAs with different biophysical properties. Our observations therefore beg an explanation at the level of whole pathways, and here we propose an energetic explanation. That is, patterns of codon bias in genes encoding functionally-related proteins influence translational efficiency in multiple components of a given pathway, thus increasing or decreasing the efficiency of translation across that pathway.

## Methods

### Datasets and the concept of Differential Entropy

Using Ensembl BioMart (http://www.biomart.org/), we acquired the entire protein coding sequences (CDS) for six organisms that occupy vital positions in the tree of life: *S. cerevisiae* S288C (March 2010), simple unicellularity, basal eukaryote; *A. thaliana* TAIR 10 (April 2011) transition to multicellularity, Plant Kingdom; *C. elegans* WS210 (November 2010), transition to simple multicellularity, Animal Kingdom; *G. gallus* 2.1 (May 2006), non-mammalian vertebrate, complex multicellularity; *P. troglodytes* 2.1 (March 2006), mammalian vertebrate, complex multicellularity; and *H. sapiens* HG19 (February 2009), highly complex multicellularity, including cognitive function.

Then, for every CDS (of which there are ∼55,000 in the human genome) we generated 20 random sequences that encode the identical amino acid sequence. This allowed us to accurately characterize the entropy that could be expected in the absence of codon bias (Table S1 contains the entropy data for every sequence in each species). We followed the randomization procedure of Itzkovitz et al. [15], who demonstrated that 20 realizations is more than adequate in this context. In passing, we wish to point out that this approach-in the absence of the downstream statistic described below-may be useful in determining ‘neutral’ rates of codon usage for molecular evolutionary studies.

We devised a new codon bias statistic–**DI**fferential **C**odon usage **E**ntropy **(DICE)** by subtracting the entropy of the observed sequence from that of the expected:
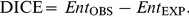
(1)


The analysis is performed strictly at the codon level, which means that any increase in regularity (detected by a reduction in information entropy) can be exclusively attributed to codon usage bias. Under this definition, coding sequences with high regularity imposed by codon bias will be awarded a strong negative value of DICE. It also means that regularities imposed by 1) repetitive tracts of amino acids, 2) disproportionate representation of low-redundancy amino acids and 3) short sequence length [16] do not distort interpretation. Because the concept of entropy is irreducible and only has meaning in the context of the sequence taken as a whole, this novel approach for measuring codon bias is in the spirit of the modern integrative systems biology paradigm.

In making the cross-species comparisons we identified orthologs (i.e. presumed similar or same function) by gene symbol. This is a relaxed criterion and may contain false positives. As a second level of quality control, when assessing the genes at the extremes of the statistical output we determined whether they were true orthologs through targeted Pubmed searches on a case-by-case basis. Additionally, the human data contain a number of coding sequences per gene. In making direct cross-species comparisons between presumed orthologs we used the human gene variant that most closely matched the gene in the compared species, as determined by sequence length.

### Shannon's Entropy and Approximate Entropy

To quantify the amount of regularity in a certain CDS, we explored two entropy metrics: Shannon's entropy and approximate entropy.

Shannon entropy provides a scientific method to express the degree of uncertainty of a probabilistic event [17]. The entropy is calculated as a product of probability and the logarithm of probability for each possible state of the targeted variable (one of four nucleic acids in our case), defined as follows [17]:
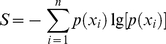
(2)where *n* is the length of sequence series and *p*(*x_i_*) is the probability of every component in the signal, satisfying the constraint Σ*p*(*x_i_*) = 1. In the DNA ‘alphabet’, *x_i_* only has four states: Adenine, Cytosine, Guanine, and Thymine; so *n* = 4 [18]. Notably, assuming equal proportions of nucleotide usage (i.e., *p*(*x_i_*) = 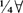 *x_i_*) it can be shown that *S* = 2.0 and any deviation from equal proportions implies *S*<2.0.

Approximate entropy (ApEn) is a non-negative number, which denotes the complexity of a sequence by measuring the likelihood of pattern occurrence [19]. Given a sequence containing *N* data points {*u*(*i*): 1≤*i*≤*N*}, the algorithm to compute ApEn proceeds as follows:

- **Step 1:** Compose the *m*-D (dimensional) vector *X*(*i*) with sequence *u*(*i*) according to its order:

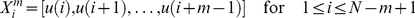
(3)where 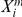 represents *m* consecutive *u* values, commencing with the *i-*th point.

- **Step 2:** Define the distance 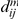 between 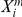 and 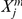 as:

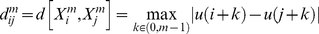
(4)
**Step 3:** For each vector 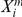, construct a measure that describes the similarity between the vectors 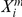 and 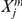:

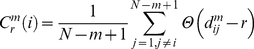
(5)where *r* represents a predetermined tolerance value, and 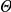
denotes the Heaviside unit step function defined as follows [20]:
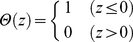
(6)

- **Step 4:** Calculate the logarithmic average over all the vectors of 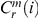
probability:

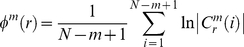
(7)
**Step 5:** The ApEn value is given by:

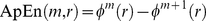
(8)Thus, ApEn of a sequence measures the likelihood that runs of patterns of length *m* that are close to each other will remain close in the next incremental comparison, *m*+1. A greater likelihood of remaining close (high regularity) produces more extreme negative ApEn values, and vice-versa. To compute entropy the data stream must be numeric. In the present study, the value *u*(*i*) was assigned to numbers 1, 2, 3 and 4 for nucleotide bases Adenine, Cytosine, Guanine, and Thymine, respectively. This choice of numeric is admittedly arbitrary but in fact has no impact on the downstream computations so long as the notation is consistent, for reasons of location and scale invariance. For example, we have performed the entropy calculations after assigning the nucleotides their molar mass in place of 1, 2, 3 and 4-and derived exactly the same results. The two parameters, *m* and *r*, must be fixed to compute approximate entropy. The values *m* = 2 and *r* = 0.15 or 0.2 are recommended [21]. In the present study, *r* was set as 0.2. However, other values did not affect the results when the DICE of a given CDS was compared with other sequences and across genomes.

### Sampling distribution of Differential Entropy

To increase our understanding of the numerical behavior that could be expected in applying DICE to real CDS, we devised a simulation schema by which six ‘master’ sequences were randomly generated with varying lengths of 300, 900, 1500, 3000, 4500 and 9000 amino acids. Then, for each ‘master’ sequence we produced 20 ‘synonymous’ DNA sequences that would code for the same sequence of amino acids yet using synonymous codons at random. The entropy, both Shannon's and ApEn, of each ‘master’ and ‘synonymous’ sequences was computed. The value of DICE was derived from the difference between the entropy of the ‘master’ sequence and the average entropy of its corresponding ‘synonymous’ sequences. Finally, the whole process was repeated 1,000 times.

For each set of ‘master’ *versus* ‘synonymous’ simulated sequences, we computed the percentage error rate as follows:
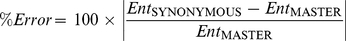
(9)


The resulting *%Error* at various sequence lengths was analyzed to identify the threshold that should be employed for a DICE corresponding to an empirical statistical significance of *P-*value<0.01. After ranking all the *%Error*, the 10^th^ ranked value, out of 1,000 simulations, was used as threshold.

### Functional Enrichment Analysis

To assess the biological relevance of the output we ranked DICE on a within-lineage basis, then pasted each list in turn into the GOrilla web tool [9]. GOrilla uses hypergeometric statistics of gene ontology terms to identify coordinated patterns of functional enrichment at the top of the list. The difference in differential codon bias – contrasting the same gene between lineages – was also explored by plotting each organism versus human and manually identifying coordinated patterns of bias favoring one of the lineages.

## Results

### Simulation results

Table 1 provides a summary of the results from the simulated datasets. Regardless of the measure of entropy used (Shannon's or ApEn), we observed a decrease in DICE as the length of the sequence increased. Because neither the ‘master’ nor the ‘synonymous’ sequences were simulated with codon bias, the decrease in DICE as the length of the sequence increased indicated that DICE provides a consistent estimate of the codon bias because its own bias tends to zero as sample size increases. The smaller variation observed for Shannon compared with ApEn can be deemed an artifact imposed by the bounded upper limit of 2.0 for Shannon's. ApEn does not suffer from such bounds. Similarly, ApEn showed an increased power to detect a significant DICE at a pre-defined statistical significance level (last two columns and last row of Table 1). These results favor the use of ApEn when computing DICE, and consequently our analysis and discussion is based on ApEn. We should point out that the two entropy measures are so highly related that the application of Shannon's entropy would highlight the same genes and biological pathways, but may assign them slightly different p-values.

**Table 1:** Differential Entropy, measured using either Shannon or Approximate Entropy (ApEn), as a function of simulated coding sequences of varying lengths.

Length, bp	Range (max – min)^A^		1% Significance Threshold^B^	
	Shannon	ApEn	Shannon	ApEn
300	0.047	0.115	0.818	3.746
900	0.018	0.039	0.340	1.155
1,500	0.012	0.029	0.231	0.759
3,000	0.006	0.014	0.145	0.395
4,500	0.005	0.010	0.097	0.292
9,000	0.004	0.006	0.059	0.166
Equation for Best Fit^C^:			65.95*x* ^−0.77^	618.40*x* ^−0.91^

A In all cases, the average Differential Entropy was within three decimal digits from zero.

B Percentage error rate threshold corresponding to empirical *P*-value<0.01

C Prediction equations (both with R^2^>99%) to identify coding sequences with statistically significant Differential Entropy (*P*-value<0.01).

### Genome-wide and lineage-specific Differential Entropy

**Figure 1:**
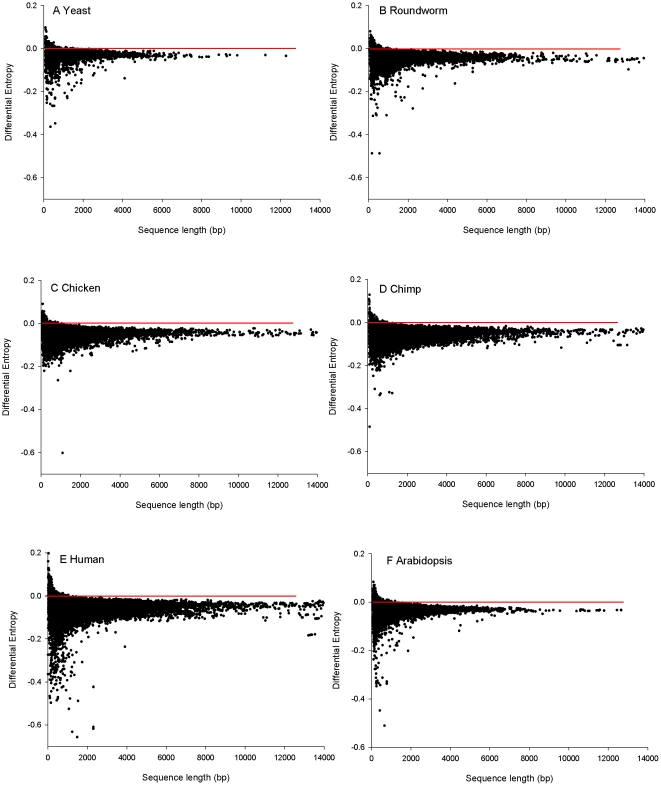
Differential Entropy: Regularity in coding sequences expressed as the difference between the observed and the randomly expected entropy. Negative values indicate sequences more regular than expected for a given amino acid sequence. The horizontal red line is positioned at zero on the y axis. All sequences below this line possess codon bias.

Table 2 provides summary statistics for the real versus the random entropy for the six species. Most of the sequences for these species possess lower entropy than random. Figure 1 illustrates the genome-wide DICE for the six species under consideration. For shorter noisier sequences the real sequence might have higher entropy (i.e. more disorder) than the simulated random sequences through chance alone. This effect disappeared with increasing sequence length as the entropy measurement of both the real and the random sequences became more robust. The distribution of the data points around the line, and the far greater mass below the line, suggests the statistic is independent of sequence length.

**Table 2:** Average Shannon and Approximate entropy (ApEn) of real and random coding sequences (CDS), and percentage of CDS where the entropy of the real sequence is less than expected by chance across the six species.

	Yeast	*C. elegans*	*A. thaliana*	Chicken	Chimp	Human
Number of CDS	6,413	27,974	32,936	18,536	30,973	56,323
Entropy of real CDS						
Shannon	1.954	1.970	1.976	1.971	1.968	1.965
ApEn	1.300	1.294	1.303	1.290	1.287	1.276
Entropy of random CDS^A^						
Shannon	1.979	1.983	1.986	1.986	1.986	1.984
ApEn	1.331	1.336	1.337	1.339	1.340	1.330
% Observed<Random						
Shannon	93.44	78.22	84.64	79.97	82.10	82.28
ApEn	97.47	99.12	98.61	99.28	99.37	98.75
% with Significant (P<0.01) Differential Entropy						
Shannon	82.55	65.28	62.01	60.86	65.18	63.43
ApEn	61.02	77.66	68.54	83.67	85.03	79.82

A For every CDS, we generated 20 random sequences that encode the identical amino acid sequence to compute average entropies.

The within-lineage functional enrichments are summarized in Table 3. Notable among these enrichments are ‘translation’ for yeast, ‘cell wall’ and ‘chloroplast’ for *A. thaliana*, ‘keratinization’ for chimp and ‘sequence-specific DNA binding’ for human.

**Table 3:** The 5 most extreme functional enrichments for each species on a within-lineage basis.

Species	Biological process	*P*-value^A^
Yeast	Translation	4.26E-90
	Regulation of Translation	4.70E-51
	Posttranscriptional regulation of gene expression	6.86E-48
	Ribosome assembly	4.74E-14
	rRNA processing	2.32E-13
C. elegans	Nucleosome organization and assembly	1.55E-17
	Protein-DNA complex organization and assembly	1.11E-16
	Body morphogenesis	1.77E-13
	Translation	5.54E-13
	Chromatin organization	2.07E-8
A. thaliana	Structural constituent of cell wall	7.75E-14
	Translational elongation	6.62E-10
	Plant-type cell wall organization	1.37E-7
	Structural constituent of ribosome	7.63E-7
	Chloroplast ribulose bisphosphate carboxylase complex	1.46E-4
Chicken	Regulation of multicellular organismal process	1.95E-7
	Sex determination	2.31E-6
	Regulation of transcription, DNA dependent	4.67E-6
	Regulation of cell differentiation	5.28E-6
	Regulation of developmental process	7.68E-6
Chimpanzee	Keratinization	2.67E-18
	Feeding behavior	5.93E-7
	Epidermal cell differentiation	2.56E-5
	Regulation of transcription, DNA-dependent	6.59E-5
	Pigment accumulation in tissues	5.47E-4
Human	Sequence-specific DNA binding activity	3.68E-11
	Hormone activity	4.24E-8
	Regulation of transcription, DNA-dependent	8.99E-7
	RNA polymerase II transcription factor activity	5.41E-6
	Epidermal cell differentiation	6.99E-5

A Adjusted P-values for the hypergeometric test obtained using the GOrilla tool (Eden *et al.*, 2009), http://cbl-gorilla.cs.technion.ac.il/.

**Figure 2:**
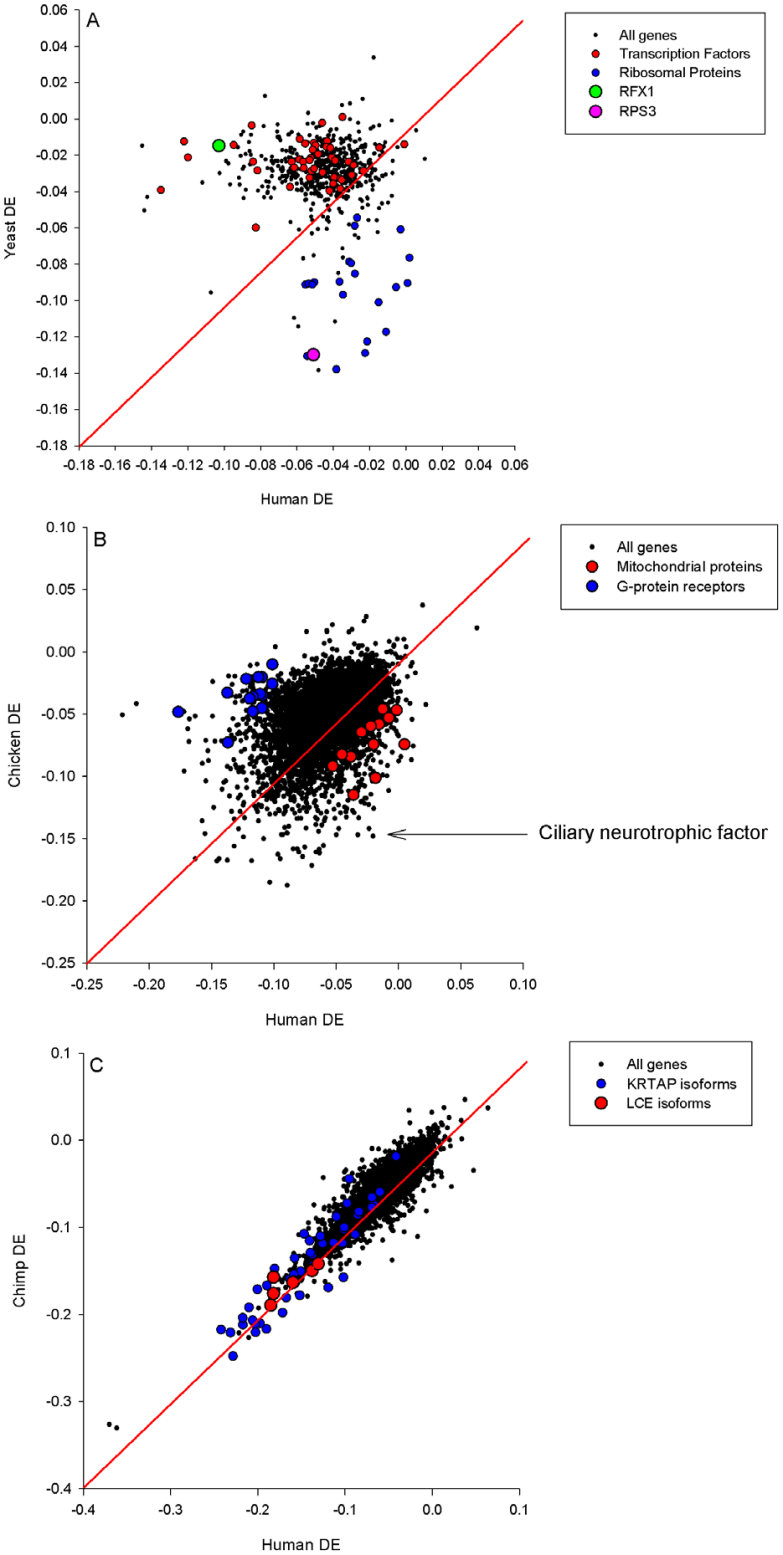
Differential Entropy in sequences from 609 orthologous proteins in humans and yeast (A). Highlighted are the ribosomal proteins (N = 23; blue), the transcription factors (N = 47; red), RFX1 (green) and RPS3 (pink). Differential Entropy in sequences from 7,902 orthologous proteins in humans and chicken (B). Highlighted are mitochondrial proteins (N = 14; red), G-protein receptors (N = 14; blue) and CNTF (Table 5). Differential Entropy in sequences from 14,182 orthologous proteins in humans and chimps (C). Highlighted are the keratin associated proteins (N = 46; blue). The diagonal red lines are 45 degree bisectors that have been placed to show the point at which there is no difference in bias between species. The perpendicular distance from the diagonal represents the extent of the difference in bias.

In specifically comparing the genes encoding the equivalent proteins in humans versus yeast (Figure 2A), humans versus chicken (Figure 2B) and humans versus chimps (Figure 2C) we noticed coordinated differences in sequences coding for functionally-related proteins. The yeast genes had a more extreme codon bias in sequences coding for ribosomal proteins (hypergeometric test *P*-value = 4.26 × 10^−90^), whereas the human genes had a more extreme codon bias for DNA binding transcription factors (hypergeometric test *P*-value = 3.68 × 10^−11^) (Table 3). The chicken genes had a more extreme codon bias in sequences coding for mitochondrial proteins (hypergeometric test *P*-value = 1.88×10^−4^), whereas the human genes had a more extreme codon bias in sequences coding for G-protein receptors (hypergeometric test *P*-value = 6.76×10^−6^) (Figure 2B). Human and chimp genes both possessed extreme codon bias in the sequences coding for the KRTAP family of proteins (Figure 2C).

**Table 4:** Codon usage in transcription factor RFX1 and ribosomal protein RPS3 in humans and yeast^A^.

		RFX1						RPS3					
		Human			Yeast			Human			Yeast		
AA	Syn.	N	PC	Prop.	N	PC	Prop.	N	PC	Prop.	N	PC	Prop.
Phe	2	20	TTC	0.900	39	TTC	0.513	7	TTT	0.714	8	TTC	0.875
Leu	6	86	CTG	0.640	81	TTA	0.370	21	CTG	0.524	19	TTG	0.632
Ile	3	22	ATC	0.909	57	ATT	0.386	15	ATC	0.533	13	ATC	0.539
Trp	1	9	TGG	1.000	4	TGG	1.000	1	TGG	1.000	0	TGG	0
Val	4	81	GTG	0.630	33	GTT	0.333	25	GTG	0.560	25	GTC	0.520
Ser	6	91	AGC	0.451	116	TCA	0.259	10	TCT	0.300	9	TCT	0.556
Pro	4	89	CCC	0.494	62	CCA	0.323	17	CCC	0.412	11	CCA	1.000
Thr	4	64	ACC	0.563	43	ACA	0.395	13	ACT	0.385	13	ACT	0.616
Ala	4	96	GCC	0.573	31	GCA	0.387	18	GCT	0.444	28	GCT	0.964
Tyr	2	33	TAC	0.818	21	TAC	0.524	6	TAC	0.667	7	TAC	0.858
Cys	2	6	TGC	0.667	13	TGT	0.538	3	TGC	0.667	1	TGT	1.000
His	2	18	CAC	0.889	15	CAT	0.667	3	CAC	1.000	2	CAC	1.000
Gln	2	105	CAG	0.905	33	CAA	0.697	8	CAG	0.875	7	CAA	1.000
Asn	2	20	AAC	0.950	75	AAT	0.587	3	AAT	0.667	5	AAC	1.000
Lys	2	29	AAG	0.828	58	AAA	0.672	20	AAG	0.700	18	AAG	0.667
Arg	6	36	CGG	0.417	24	AGA	0.500	18	CGG	0.333	20	AGA	0.900
Asp	2	25	GAC	0.920	29	GAT	0.621	8	GAC	0.500	9	GAC	0.778
Glu	2	52	GAG	0.885	39	GAA	0.741	18	GAG	0.611	22	GAA	1.000
Gly	4	79	GGC	0.684	22	GGC	0.364	23	GGC	0.391	17	GGT	1.000
Met	1	18	ATG	1.000	16	ATG	1.000	16	ATG	1.000	6	ATG	1.000

A For each amino acid (AA) the number of synonymous (Syn.) codons is given. For each protein sequence, three values are given: the number (N) of occurrences of each AA, the preferred codon (PC) and the proportion (Prop.) in which the PC is used.

### Codon bias shifts from translation to transcription

The comparison between the codon bias of the genes encoding the ∼600 orthologous proteins common to humans and yeast yielded dramatic and divergent patterns of codon bias, favoring ribosomal proteins in yeast and transcription factors in humans (Figure. 2A).

This is further illustrated in Table 4, which provides a detailed dissection of the differential codon usage between two genes in humans and yeast, encoding the proteins RFX1 and RPS3. The transcription factor RFX1 is a member of the regulatory factor X gene family known to be conserved throughout evolution from yeast to humans [22]. The ribosomal protein S3 (RPS3), originally identified as a component of the small ribosomal subunit where it is involved in protein synthesis [23], has subsequently been shown to participate in many processes including the oxidative stress pathway [24], NF-kappaβ complex [25] and the maintenance of genomic integrity [26].

**Table 5:** Codon usage in the protein CNTF in chicken and humans^A^.

		CNTF					
		Chicken			Human		
AA	Syn.	N	PC	Prop.	N	PC	Prop.
Phe	2	2	TTC	0.667	4	TTC	0.571
Leu	6	25	CTG	0.807	8	CTG	0.307
Ile	3	3	ATC	1.000	5	ATC	0.417
Trp	1	2	TGG	1.000	4	TGG	1.000
Val	4	7	GTG	0.636	4	GTG	0.500
Ser	6	6	AGC	0.462	4	TCT	0.308
Pro	4	4	CCC	0.400	3	CCA	0.429
Thr	4	4	ACC	0.500	6	ACC	0.500
Ala	4	12	GCC	0.462	7	GCT	0.467
Tyr	2	3	TAC	1.000	3	TAT	0.600
Cys	2	1	TGC	1.000	1	TGT	1.000
His	2	4	CAC	1.000	9	CAT	0.900
Gln	2	10	CAG	1.000	8	CAG	0.667
Asn	2	1	AAC	1.000	6	AAC	0.750
Lys	2	3	AAG	1.000	8	AAG	0.889
Arg	6	9	CGG	0.474	5	CGT	0.417
Asp	2	9	GAC	0.819	6	GAC	0.600
Glu	2	17	GAG	1.000	10	GAG	0.714
Gly	4	9	GGC	0.692	4	GGG	0.400
Met	1	4	ATG	1.000	5	ATG	1.000

A For each amino acid (AA) the number of synonymous (Syn.) codons is given. For each protein sequence, three values are given: the number (N) of occurrences of each AA, the preferred codon (PC) and the proportion (Prop.) in which the PC is used.

The evolutionary conservation of RFX1 contrasts with the differential codon bias observed between humans and yeast, with a much larger codon bias observed in the human gene (Table 4). For instance, phenylalanine (Phe), for which two synonymous codons exist (TTT and TTC), is preferentially encoded by TTC in humans in 18 out of 20 instances (binomial *P*-value = 2.00×10^−5^) while no codon preference was seen in yeast (49% TTT and 51% TTC).

Similarly, the multi-functionality and ubiquitous role of RPS3 in protein synthesis makes the codon bias observed in the corresponding yeast gene most remarkable. For instance, Phe, shows a preferential codon usage with TTC used in 7 out of 8 occasions in yeast (binomial *P*-value = 3.91×10^−3^), while no significant codon bias was observed in humans.

Although human and yeast RFX1 are considered orthologous [27] the human sequence is actually more regular at the amino acid level, possessing regions of biased amino acid composition. DICE does account for differences in amino acid sequence composition, however it might be argued that RFX1 could be misrepresentative of our approach.

A better illustration of the power of DICE to quantify codon bias irrespective of amino acid bias is our comparison of the human and chicken coding sequences for Ciliary Neurotrophic Factor (CNTF), a hormone and nerve growth factor that promotes neurotransmitter release and neurite outgrowth. In this comparison (Table 5), the two species had much more similar amino acid sequence composition, and the extreme DICE in the chicken was evidently driven by stronger patterns of codon bias in chickens than in humans. For example, in chicken CNTF, 10 of the 20 amino acids are exclusively encoded by a single codon, as opposed to only three of 20 in the human ortholog.

### The bias towards keratinization in humans and chimps

The comparison between the genes encoding the ∼14,000 orthologous proteins common to humans and chimps revealed that genes relating to keratinization, well represented by the LCE family of proteins, possessed extreme codon bias in both humans (*P* = 1.79×10^−19^) and chimps (*P* = 4.68 × 10^−18^; Table 3). Manual inspection of the ranked list determined that keratin-associated proteins (KRTAP) were also highly enriched near the top of both lists, but for unknown reasons were not identified by GOrilla (blue dots in Figure 2C). Consequently, the true hypergeometric enrichment for ‘keratinization’ is likely to be much stronger than the *P*-values reported above.

Although the KRTAP proteins are high in the amino acid cysteine and possess a (albeit weak) repeating structure, this does not drive the DICE output, as the alpha keratins also possess large repeating blocks of amino acids (in this case glycine) yet were not awarded an extreme score.

## Discussion

In this report we describe and implement a new approach for measuring differential codon bias. Codon bias has previously been measured using bioinformatics methods such as the frequency of optimal codons [28] and the codon adaptation index [29], which are used to predict protein expression levels. Methods from information theory such as the effective number of codons [30] and Shannon entropy [17] have been used to measure codon usage evenness. However, these approaches are influenced by the length of the coding region analyzed [31], which complicates attempts to fairly compare genes and gene families.

Our approach provides differential codon bias measurements for each gene from each of a number of organisms in a manner that allows direct comparisons between genes and sets of genes, both within and between species and lineages. On a within-species basis, the output can be ranked and objectively assessed for functional enrichment. In the analyses where we directly compared the orthologs from two species (Figure 2), we were in effect computing the difference in the differential codon bias, or the ‘differential differential codon bias’ (from now on referred to as the difference in the differential codon bias). This is an important distinction, because while the codon bias in the ortholog from each species may not be particularly pronounced or noteworthy when compared to other genes *within* that lineage, the *difference* in its properties *between* the two species can still be substantial. The discussion that follows relates to the combination of these analyses.

Unsurprisingly, we found that the vast majority of CDS in all species were more regular than random (Table 2; Figure. 1), reflecting the ubiquitous presence of codon bias documented previously by many authors. More unexpected, however, were the outputs of the pathway enrichment analyses. These analyses-built on hypergeometric-based considerations of Gene Ontology annotations - have only become available in the post-genomic era. After ranking the differential codon bias output on a within-lineage basis, we detected very strong signals suggesting that entire batteries of functionally-related genes have been subject to selection for extreme differential codon bias in a lineage-specific manner.

In ascending the scale of phenotypic complexity from yeast through roundworm to humans, we found enormous enrichment of first translation (P = 4.26 × 10^−90^), then DNA packing (P<1.55 × 10^−17^), through to regulation of transcription (P<3.68 × 10^−11^) (Table 3). We speculate that the observed decrease in functional enrichment as one ascends phenotypic complexity may reflect the greater number of competing demands imposed by selection on those more complex lineages, such that the strength of selection for translational efficiency on any one biological pathway or process is diluted by pressure on other pathways. This reasoning implies that the evolution of phenotypic complexity involves greater interdependence of different pathways and processes, which seems logically correct but perhaps difficult to prove.

Our comparison between the codon bias of the ∼600 orthologous proteins common to humans and yeast yielded dramatic and divergent patterns of codon bias, favoring relatively more bias in genes encoding ribosomal proteins in yeast, and relatively more bias in genes encoding transcription factors in humans (Figure. 2A). We hypothesize that the relative bias in transcription factors in humans versus yeast either 1) underscores the importance of transcriptional control in the evolution of more complex eukaryotes or 2) underscores selection in humans for transcriptional speed and efficiency. Further investigations based on these findings may shed light on macro scale genome-to-phenome relationships, including a possible contribution to the debate on the c-value enigma.

In analyzing *A. thaliana*, our sole representative of the Plant Kingdom, we noted that two of the top five functional enrichments related to cell wall formation and chloroplast function, both of which are unique and diagnostic of plant anatomy and physiology. Similarly, the comparison between the genes encoding the ∼14,000 orthologous proteins common to humans and chimps revealed extreme codon bias in both species for the LCE and KRTAP families of proteins, which drive the formation of hair (Figure. 2C). Hair is a tissue unique to the mammalian lineage [32], plays a crucial role in the retention of endothermic heat and contributed to the rapid rise of mammals as the dominant terrestrial vertebrate [33]. Previous research has classified the KRTAP by their amino acid composition [32], [34]. However, none have documented the extreme codon bias existing for these proteins relative to the rest of the proteins and even the alpha keratins, the other major component of hair [35].

Neither transcription factors nor the hair-related genes have previously been documented as possessing extreme codon bias characteristics in humans. These observations are consistent with our hypothesis that extreme codon bias is particularly associated with processes unique to – or diagnostic of – a given lineage in the eukaryotic tree of life.

Along the same lines, the comparison of orthologous proteins in humans and chicken identified a particular subset of mitochondrial proteins (TXNDC17, NDUFS5, NOX1, GSR, NQO2 and four sub-components of NADH1, which is the ‘gate-keeping’ enzyme of oxidative phosphorylation: NDUFB6, NDUFA2, NDUFB2 and NDUFAB1) as possessing relatively extreme codon bias in chicken (Figure 2B). Mitochondria are considered to be under extreme selective pressure in birds because of the energetic and aerodynamic demands associated with flight [36]–a process that has led to various adaptations at the physiological level such as an increase in respiratory efficiency and reduced free radical leakage [36]. We hypothesize that the DNA sequence characteristics we have identified here may similarly reflect the flight-based energetic adaptations in the avian lineage, but manifest at the molecular (i.e. translational) rather than physiological level.

Although we have focused our analysis on statistical enrichment of *groups* of genes and are wary of inferring meaning to isolated cases, we do wish to draw attention to one gene, as an interesting case in point. Ciliary Neurotrophic Factor (CNTF) shows the greatest difference in differential codon bias out of the 7902 orthologous sequences that are common to chicken and humans (Figure 2B). According to the White Paper outlining the scientific rationale for sequencing the chicken [37] “chickens have a remarkable capacity for hair cell regeneration that results in spontaneous recovery from forms of deafness…that are permanent when they occur in humans…” Intriguingly, experimental CNTF infusion has been shown capable of restoring auditory function following chemically-induced deafness in guinea pigs [38]. While the control of the expression of the CNTF protein is presumably multi-faceted, we hypothesize that the predicted reduced energetic demands for translating CNTF protein in chicken – as a consequence of extreme codon bias – facilitates translation of the protein *in the key chicken tissues at the key times*, thereby contributing to the species' unusual regenerative capacities.

In addition to extreme differential codon bias (within a species), and extreme differences in differential codon bias (between species), we were also interested in exploring whether there were any protein coding sequences in the data that did *not* show differences in differential codon bias between the various lineages. That is, have some sequences been impervious to (evolutionary) modulation in codon bias, for whatever reason? To home in on this question, we focused on the human, chimp and chicken data, thereby exploring the issue in the specific context of vertebrates. The exclusion of yeast, Arabidopsis and roundworm enabled a high enough density of data to make the comparison statistically meaningful. We discovered that this approach strongly enriched for ribosomal proteins and a large number of mitochondrial proteins. This implies that while codon bias is apparent in these sequences, the bias is not variable within the vertebrates – that is, they are similarly biased. This is perhaps consistent with the observations that ribosomal and mitochondrial proteins are coordinately and constitutively highly expressed across a range of species and circumstances.

Nevertheless, there is also an implication that certain aspects of the mitochondrial energy transfer process are more amenable to modulation than others. The codon bias data from this analysis showed that in a representative from a phylogenetic group possessing high-performance energetics (the avian lineage), the DNA sequences coding for most mitochondrial proteins have similar codon bias to those in other vertebrates. But at the same time we observed that a specific subset of genes coding for mitochondrial proteins possess an extreme differential codon bias in the chicken compared with humans. These genes – particularly those encoding the various subunits of the NADH1 complex that catalyzes the entry point to oxidative phosphorylation – arguably represent possible targets for rational attempts to increase energetic efficiency in other organisms. Further insights might be gained by examining the DNA sequence encoding the mitochondrial machinery in an elite avian flight performance model, such as the hummingbird.

Our method for assessing codon bias (DICE) has not been formally tested against competing codon bias metrics. We presume that existing metrics would identify exactly the same macro evolutionary patterns documented herein, and it is not clear to us why they have remained undetected. One explanation could relate to previous emphasis on prediction of protein expression levels rather than genome-wide functional enrichments, especially as the latter have only recently become available.

### Caveats

We wish to flag a couple of caveats associated with this analysis and its biological interpretation. Firstly, while we have interpreted extreme differential codon bias in energetic terms, codon bias can also arise for other reasons – some of which are adaptive and some of which are neutral. For example, codon bias may arise as a simple artifact of history, following duplication or some other expansion events from a smaller piece of ancestral sequence. Such expansion processes will duplicate the codon bias of the original sequence which necessarily enforces sequence regularity, in the absence of any energetic reasoning. Other possible non-energetic explanations include impact on amino acid hydrophilicity [39], nucleotide mutation bias and regional differences in nucleotide composition across the genome [40], impact on splicing [41], impact on mRNA folding [42] and the impact of random genetic drift [43].

Fundamentally, however, it seems unlikely that these alternative explanations can adequately account for the consistent functional enrichment scores detected. Our explanation rests on the observed enrichment for *whole pathways* or *processes*, not individual molecules. For example, non-energetic hypotheses could potentially explain why the LCE proteins possess extreme codon bias in mammals. But it stretches credibility that these same non-energetic hypotheses also explain the KRTAP gene sequences, given the two groups of sequences have an independent evolutionary trajectory: a PHI-BLAST search (http://www.ebi.ac.uk/Tools/sss/psiblast/) failed to find any significant relationship between the KRTAP and the LCE family of proteins. In addition, they occupy a different part of the genome and the physical and chemical behaviour of the messenger RNAs is quite different. What binds the observation is that they encode proteins that represent different components of the same biological pathway, that of ‘keratinisation.’ The implication is clear: the *pathway itself* must have been selected for.

Our current belief is that approaches based on comparisons of genome-wide codon bias lend themselves to macro evolutionary analyses. This is because the larger the phylogenetic distance between comparison species, the more robust the numerical signal for differential codon usage. However, this presents a challenge to functional interpretation. As the distance between the compared species increases so too does the extent and number of phenotypic differences. This makes it difficult to functionally interpret the output.

### Conclusions

We have systematically quantified codon bias in several key eukaryotes. In doing so, we have identified lineage-specific patterns of codon bias that have not previously been reported. Some of these only became apparent through comparisons between species. Our working hypothesis is that patterns of extreme codon bias highlight molecules and pathways from a particular lineage that have been given energetic priority (assuming bias towards preferred codons) through natural selection. These patterns identify genes and gene families unique to, or having particular relevance in, a given lineage (such as hair in mammals, and cell walls in plants). Our hypothesis is supported by functional enrichments for entire pathways or processes, not merely individual molecules. These enrichments are built on observations of cohorts of DNA sequences that possess independent evolutionary histories and quite different messenger RNA characteristics.

## Supporting Information

Table S1 **For all coding sequences for the six species explored, this file provides the entropy of real sequences and the average entropy of 20 random sequences coding for the same amino acids.** These data allow for the reconstruction of all the analyses.
(XLS)
